# Cochlea implantation in patients with superficial hemosiderosis

**DOI:** 10.1007/s00405-021-07198-2

**Published:** 2021-12-20

**Authors:** E. Artukarslan, F. Matin, F. Donnerstag, L. Gärtner, T. Lenarz, A. Lesinski-Schiedat

**Affiliations:** 1 Department of Otorhinolaryngology, Hannover Medical School, Hannover, Germany; 2 Department of Neuroradiology, Hannover Medical School, Hannover, Germany

**Keywords:** Superficial hemosiderosis, Cochlear implant, Cochlear spiral ganglion neurons

## Abstract

**Introduction:**

Superficial hemosiderosis is a sub-form of hemosiderosis in which the deposits of hemosiderin in the central nervous system damage the nerve cells. This form of siderosis is caused by chronic cerebral hemorrhages, especially subarachnoid hemorrhages. The diversity of symptoms depends on the respective damage to the brain, but in most of the cases it shows up as incipient unilateral or bilateral hearing loss, ataxia and signs of pyramidal tracts. We are investigating the question of whether cochlear implantation is a treatment option for patients with superficial hemosiderosis and which strategy of diagnostic procedure has to be ruled out preoperatively.

**Materials and methods:**

In a tertiary hospital between 2009 and 2018, we examined (*N* = 5) patients with radiologically confirmed central hemosiderosis who suffered from profound hearing loss to deafness were treated with a cochlear implant (CI). We compared pre- and postoperative speech comprehension (Freiburg speech intelligibility test for monosyllables and HSM sentence test).

**Results:**

Speech understanding improved on average by 20% (monosyllabic test in the Freiburg speech intelligibility test) and by 40% in noise (HSM sentence test) compared to preoperative speech understanding with optimized hearing aids.

**Discussion:**

The results show that patients with superficial siderosis benefit from CI with better speech understanding. The results are below the average for all postlingual deaf CI patients. Superficial siderosis causes neural damages, which explains the reduced speech understanding based on central hearing loss. It is important to correctly weigh the patient's expectations preoperatively and to include neurologists within the therapy procedure.

## Introduction

**Fig. 1:**
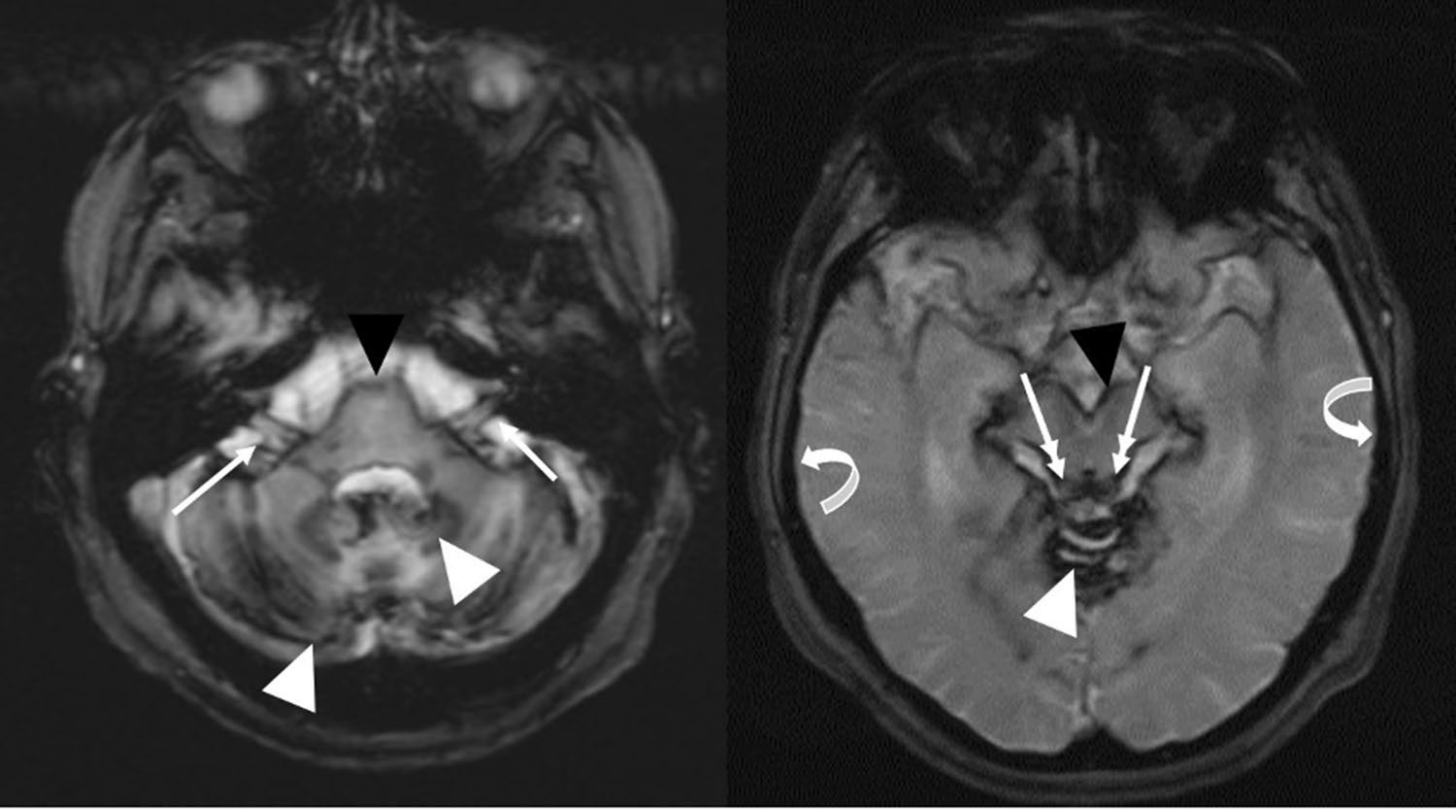
Axial T2*-weighted MRI of Pat XX with superficial siderosis reveals diffuse hypointensity outlining the cortex and nerval tissue in the posterior fossa. **A** Siderosis of the cranial nerves VII, VIII, IX from their pontine emergence up to the entry in the inner auditory canal (white arrows) and siderosis the cerebellar cortex (white arrow heads). **B** Siderosis of the quadrigeminal plate (double white arrows) and the upper cerebral vermis (white arrow heads) with sparing of the cortex of both temporal lobes (curved white)

Superficial hemosiderosis (SH) is a rare progressive disease of the central nervous system (CNS). SH is resulting from subarachnoid bleeding primary build-up of the hemosiderin in the leptomeninges, the cranial nerves, spinal cord and in the subpial tissue [1–6]*.* The cause of this form of accumulation is chronic bleeding, wherein the hemosiderin acts cytotoxically on the surrounding subarachnoid tissue [5]*.* The onset of clinical symptoms ranges from 14 to 77 years with a male-to-female ratio of 3:1 [4, 7]. The causes of bleeding are in up to 50% of the cases idiopathic where no bleeding source is found and secondary forms, where a bleeding source can be detected [4, 6, 8]. The main causes of bleeding are head trauma, aneurysm, neoplasm and neurosurgical procedures [5]. In a variably number of superficial siderosis a intraspinal fluid-filled collection of variable dimensions is found [8]. Symptomatically, the SH manifests itself in more than 95% of cases with uni- or bilateral sensorineural hearing loss (SNHL), in 88% of cases with cerebellar ataxia and in 76% of cases with pyramidal signs. Other symptoms include dementia, anosmia and anisocoria in 24%, 17% and 10% [2, 9]. For diagnosis of SH, the magnetic resonance imaging (MRI) is to date the gold standard (Fig. 1). The gradient-echo T2* and spin echo T2-weighted are the most sensitive sequences for hemosiderin detection in the CNS marked as low-density regions [6, 10].

Pathophysiologically, the Bergmann glia absorbs the iron from the CNS. Stored in the form of ferritin, the hemosiderin is then broken down. Once the binding capacity has been exhausted, the iron ions accumulate and due to the associated lipid peroxidation, parenchymal and axonal damage to the nerves is seen. The cerebellum is a predilection site for the hemosiderin, presumably due to the presence of Bergmann glia in the upper layers. The N. VIII (vestibulocochlearis nerve) is also probably a preferred site because the long course in the pontine cistern and the resulting contact with the hemosiderin is probably higher. In addition, the N. VIII has a long glial segment and is therefore more vulnerable to the accumulation of hemosiderin [4, 5, 7].

The treatment of SH depends on the cause and remains challenging. First of all, bleeding sources should be detected and treated with surgical ablation [5]. If the origin of the SH despite extensive imaging work-out remains unknown a medical treatment with iron chelators could be considered [11]. Due to the frequent finding of intraspinal fluid-filled collection and their associations with intracranial hypotension [8, 12], an epidural blood patching may be an individual therapy option [13]. Under computer tomographic (CT) scan guidance, an 18-gauge Tuohy needle is inserted into the epidural space via a median or paramedian interlaminar approach. The epidural position of the needle is confirmed with an injection of 1–2 ml of iodine containing contrast media and subsequently, patient´s blood is injected through the needle. A second CT scan confirms the peridural distribution of the blood in the spinal canal. The patient remains in prone position for at least four hours and bedrest up to the next day.

While uni- or bilateral SNHL affects about 95% of patients with SH, hearing aids (HA) are means of choice for mild to moderate SNHL. In cases of progressive SNHL to severe-profound hearing loss (HL) where acoustic amplification is no longer sufficient, cochlear implantation (CI) is indicated. Despite retrocochlear genesis of the HL, the literature describes in the majority of cases a sufficient outcome in hearing with CI with 44 cases [6, 9, 14–17].

**Fig. 2:**
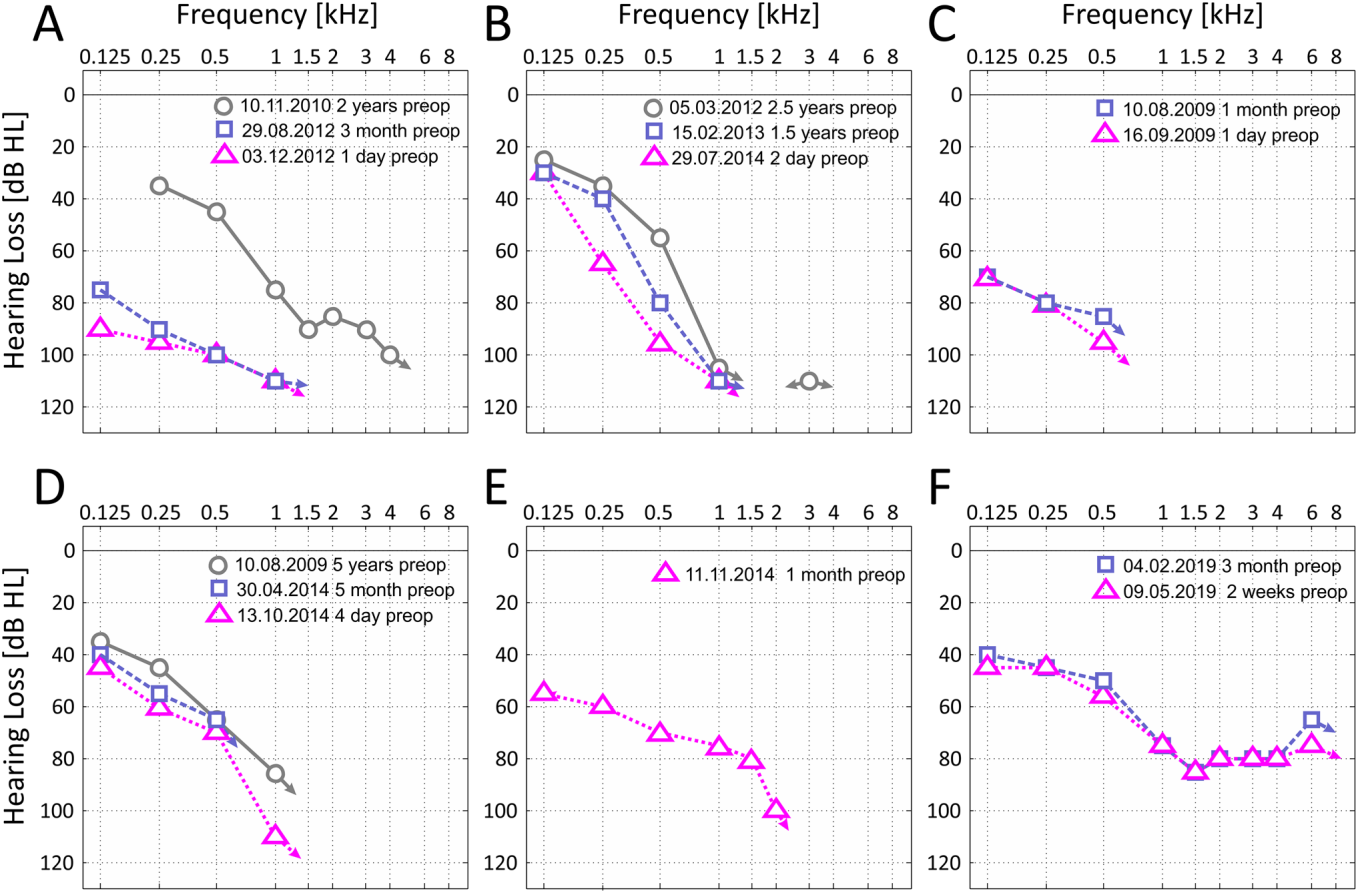
The figure shows preoperative audiograms of all our five patients. **A** Case 1; **B** Case 2; **C** Case 3 left side; **D** Case 3 right side; **E** Case 4; **F** Case 5

Between 2009 and 2018, ten patients with radiologically confirmed central hemosiderosis presented at our tertiary care hospital. Of these ten patients, five cases who suffered from profound hearing loss to deafness were treated with a cochlear implant (CI), four uni- and one bilaterally (Fig. 2). Another two patients with profound hearing loss decided against a treatment with cochlear implants and another two patients with hemosiderosis were diagnosed with a moderate hearing loss and could be fitted with hearing aids. The last patient out of the ten known cases with a profound hearing loss underwent the blood patch therapy and showed a slow progression of his hearing loss.

In this retrospective study, we showcase five patients who were treated with CI. One patient was blood patched before the CI. We report about the CI outcomes of five patients and whether the blood patch therapy had an influence on the SH symptoms. Based on pre-, intra- and postoperative measurements, we analyze the genesis of the HL.

The ethic approval for this retrospective study was pronounced positively from the responsible ethics committee (number 1897–2013).

## Case presentations

### Case 1

A 68-year-old woman was referred to our clinic in 2012 with deafness on the right and profound SNHL on the left side, ataxia and vertigo. She reported about gradual progressive HL for six years and insufficient acoustic amplification using HA. Cognitive deficits, pyramidal signs or other symptoms associated with SH were not detected. She had a history of cerebellar infarction in 2006. In 2011, the diagnosis of SH and cerebellar worm atrophy was detected with MRI. In angiography, no source of bleeding could be detected.

The audiological and neurootological evaluation revealed (Table 1) deafness on the right side and profound SNHL in the high frequencies and residual hearing in the low frequencies on the left side. In the Freiburg Monosyllabic Word Test (FMWT) in quiet at 65 dB sound pressure level (SPL) under best aided condition, she showed 0% recognition on the right side and 10% recognition at 80 dB on the left side. Auditory brainstem response (ABR) did not record any responses at maximum intensity of 95 dB nHL on both sides. Transient evoked otoacoustic emissions (TEOAE) were not present bilaterally. The transtympanic promontory electrical stimulation (tt-PES) elicited a sound sensation on both sides. The vestibular examination showed a canal paresis on the left side in the caloric test and a vestibular and visual deficit in the posturography. The CT of petrous bones showed no abnormalities. The CI surgery was conducted in 2012 on the right side with a CI24RECA electrode that was fully inserted using a round window approach (RWA). Intra- and postoperatively, no complications were reported.

**Table 1:** Various audiological data collected pre- and postoperatively, symptoms, age and the laterality of the CI

	Gender	Age of first subj. HL (years)	Age at implantation (years)	Laterality of CI	Symptoms	Pre-OP FMWT (%)	POFMWT (%) after one year	Pre-OP HSMwn (%)	POHSwn (%) after one year	Reference due to our clinic with no SH in 2019 (%)
Case 1	F	68	68	R	Ataxia, Vertigo, HL	0	0	Not measured	0	60
Case 2	F	52	55	L	HL, Tinnitus	0	35	Not measured	47	60
Case 3	M	20	59, 66, 67	L, R, R	HL	0L, 5R	30R, 35 L	Not measured	53R, 83L	60
Case 4	F	66	73	L	HL, Tinnitus	25	35	30	28	65
Case 5	M	52	56	L	HL, Tinnitus	25	25	0	63	65

*HL* hearing loss; *CI* cochlea implant; *pre-OP FMWT* preoperative Freiburger monosyllabic word test; *POFMWT* postoperative Freiburger monosyllabic word test; *Pre-OP HSMwn* preoperative Hochmair–Schulz–Moser test without noise; *POHSMwn* postoperative Hochmair–Schulz–Moser test without noise; *R* right; *L* left; *M* male; *F* female

Intraoperatively, electrically evoked compound action potential (ECAP) and electrically evoked stapedius reflexes (ESR) were measured using the clinical software CustomSoundEP. ECAP responses were successfully recorded via the AutoNRT (automatic neural response telemetry) task over the whole electrode array with regular morphology. Also, ESR were elicited successfully. The Hochmair–Schulz–Moser test (HSM-Test) showed no speech understanding pre- and postoperative. ECAP responses were still detectable on each of the 22 electrode contacts. All AutoNRT traces were checked according to Gärtner et al. [18]. Only minor corrections were necessary. ECAP thresholds were found in between 174 and 210 CL (at 25 µs pulse duration) corresponding to 10.1 and 19.4 nC, respectively. The slope of the ECAP amplitude growth function (AGF) was mostly below 1 µV/CL, and hence very shallow comparing to normal CI users. Postoperatively in the follow-ups up to 13 months, the FMWT showed no improvement in speech recognition with CI.

### Case 2

In 2012, a 55-year-old female patient presented at the clinic with bilateral progressive profound SNHL for three years and bilateral tinnitus for one year. She used HA for three years but was no longer able to benefit from them despite optimization. She had a history of meningitis (unknown age). During the examination in 2012, a SH was diagnosed on the MRI in the area of the cerebellum. She was provided with a blood patch by the neuroradiology. Since there was no improvement in hearing after the treatment with blood patch, an audiological and radiological examination was carried out in 2014 (Table 1).

In the ABR, there were no stimulus responses on both sides at 95 dB nHL. No TEOAE were detectable bilaterally. The tt-PES elicited a sound sensation on both sides. Posturography showed a vestibular deficit and the caloric test normal responses on both sides. The CT scan revealed a normal configured temporal bone. CI on the left side was performed using a MED-EL SYNCHRONY FLEX24, which was fully inserted via RWA. Intraoperatively, ECAP and ESR were measured using the clinical software MAESTRO. ECAP responses were tried to record via the ART (auditory nerve response telemetry) task on three different electrode contacts (apical (E3), medial (E7), and basal (E11)). Only responses on E3 with unusual morphology (double P peak at latencies of about 400 and 600 µs) were obtained. ESR was elicited successfully on electrodes E1, E4, E8, and E12. Postoperatively, the patient complaint about vertigo on the first day and was treated using intravenous steroids and reported about an improvement of the vertigo symptoms on the fourth postoperative day. The postoperative speech perception outcomes with CI are 35% after one year. At that time, ECAP was measured up to the loudest acceptable presentation level (LAPL) which was about 28 nC. A hearing sensation was elicited on all electrodes but the most basal (E12). ECAP responses could not be retrieved.

### Case 3

In 2009, a 59-year-old male patient was referred to the clinic with a progressive SNHL on both sides since 1970. Other otological symptoms, cerebellar ataxia, pyramidal signs were not reported. He was insufficiently fitted with a HA on the left side. He reported about a peripheral schwannomatosis located on his shoulder. MRI showed SH in the area of the lamina tecti and the cerebellar upper worm, no vestibular schwannoma on both sides.

The CI pre-examination took place in the same year and is partly listed in Table 1. The ABR showed no response on both sides at 90 dB nHL. There were no TEOAE on both sides and the tt-PES elicited a sound sensation on both sides. Posturography showed a vestibular deficit and the caloric test a hyporesponsiveness on the right side without nystagmus. The CT examination showed a regular temporal bone. The patient received a CI on the left in 2009. The MED-EL SYNCHRONY FLEX28 electrode was fully inserted via RWA. There were no complications intra- and postoperatively. The intraoperative ART and ESR were normal. During the six-month follow-up, the patient's speech understanding with CI improved to 70% in FMWT in quiet at 65 dB SPL dB on the left.

During the follow-ups, the patient had a progression of the SNHL in the low frequencies on the right side. In 2014 the audiological examination revealed deafness in the high frequencies and profound SNHL in the low frequencies and the patient asked for a CI on the right side. Since 2009, the patient did not develop new otological symptoms, cerebellar ataxia or pyramidal signs. Except for a canal paresis on the right side in the caloric test, the audiological examination revealed no new findings. He received a CI MED-EL SYNCHRONY FLEX28 on the right with full insertion via RWA. There were no complications intra- and postoperatively. The ART and ESR were normal.

In 2016, the patient complained about pain above the CI device on the right side and tinnitus on the right side. Based on these complaints he did not wear the CI on the right. In addition, he reported about increasing gait and posture insecurities for a few months. The patient had a speech recognition score of 30% on the right ear, 45% on the left ear and 35% on both ears in FMWT at 65 dB SPL. A soft tissue contact of the CI electrode at the mastoid cavity was seen in the CT imaging. A CI revision on the right was performed in 2017 without any intraoperative complications. Intraoperative ART measurements did not show any ECAP response on the electrodes chosen for stimulation (E1, 3, 7, 11). The reason for that was clipping of the implant amplifier at relatively low stimulation charges, 20 nC (E3), 30 nC (E7), and 36 nC (E11). For the sake of time, recording parameters were not further optimized. ESR measurements were not performed intraoperatively. During the initial fitting, one month after surgery, ART did not reveal responses up to 28.5 nC (LAPL). MCLs were between 19 and 28 nC. The CI outcomes of both sides are 30% on the right and 35% on the left side after one year.

### Case 4

In 2014, a 73-year-old female patient with progressive SNHL on both sides for seven years and tinnitus for four years was referred to the clinic. No other otological symptoms were reported. She was provided with HA on both sides since 2007 without benefit at the time of presentation. Due to her polyarthrosis of the hip and knee joints, she had gait insecurities. The MRI showed a SH with punctum maximum on the cerebellum upper worm and tectum mesencephali left with affected nerve structures of the internal auditory canal and cerebellum bridge angle.

The audiogram showed profound SNHL on the right and deafness on the left side (Table 1). In the ABR, there were no stimulus responses on either side up to 95 dB nHL and no TEOAE on both sides. The tt-PES elicited a sound sensation on both sides. No posturography was performed because of the patients’ polyarthrosis. The caloric test showed normal responses on both sides. The CT examination showed regular structures of the temporal bone. The CI on the left (MED-EL SYNCHRONY FLEX28) was fully inserted via RWA in 2014. Intraoperatively, ECAP responses were tried to record via the ART task on seven different electrode contacts (E1, E2, E5, E7, E8, E9, E10). Up to a stimulation level of 36 nC, no response was obtained. Neither, ESR could be elicited up to 35 nC on electrodes E1, E4, E8, E11, and E12). No complications were reported intra- and postoperatively. Initial fitting was done six weeks after CI. Hearing sensation was elicited on each of the 12 electrode contacts. At that time, no ECAP responses could be recorded up to the LAPL of about 28 nC. The speech understanding outcomes with CI are 35% after one year.

### Case 5

A 56-year-old male patient with Marfan syndrome was referred to the clinic in 2019 with bilateral progressive SNHL and tinnitus since 2015. Despite an initial benefit from HA use since 2015 and regular optimizations the prosthesis did no longer provide sufficient acoustic amplification. He reported no other otogenic symptoms. No histories of head trauma, neurosurgical procedures or chronic bleeding were reported.

During the CI pre-examination in 2019, a MRI revealed a SH in the subarachnoid space.

The audiogram showed a moderate to profound SNHL in the right ear. A moderate SNHL in the low frequency range and a profound SNHL in the middle and high frequency range were shown on the left side (Table 1). At 95 dB nHL, the ABR showed no stimulus responses on either side. The TEOAE were not present on both sides. The tt-PES was only testes on the left and elicited a sound sensation. The posturography revealed a somatosensitive and vestibular deficit. The caloric test showed are flexia on both sides. Based on the results, he was implanted on the left in 2019 with a MED-EL FLEX26 electrode with full insertion via RWA. ECAP measurements were performed using the AutoART task within the clinical software MAESTRO and responses were observable on different electrode contacts (E2, E4, E7) with normal morphology and latencies. ESR was tried to elicit without success on electrodes E1, E4, E8, and E12 up to a stimulation level of about 40 nC. There were no complications intra- and postoperatively. At initial fitting, one-month postop, regular ECAP responses were recordable on all 12 electrode contacts via AutoART. ECAP AGF slope was very shallow ranging between 4 and 17 µV/nC. The one-year follow-up speech comprehension results are 25%.

## Discussion

SH is a rare disease of the CNS with an estimated incidence of 0.15% [19]. In most cases, SH is diagnosed by coincidence due to its cardinal symptom of bilateral progressive SNHL and disproportionately bad speech understanding with hearing aids because of the progressive neural damage. The literature describes symmetrical HL, beginning with the loss of high frequencies and progressing over all frequencies [6, 14, 20, 21]. The progression of symptoms especially HL varied in our patients, while patient no. five had deafness in the left ear within one year, patient no. three reported HL that had been slowly progressing since the 1970s (Table 1). In our series of patients, the majority showed a bilateral severe to profound HL with rapid progression to at least unilateral deafness on one ear disproportionately bad and speech understanding with hearing aids. Three patients in our case series reported about tinnitus as another otogenic symptom and vestibular deficits were seen in the preoperative posturography of most of our patients. Cerebellar ataxias were neither historically nor clinically diagnosed in our patients. The third cardinal symptom that is associated with SH—pyramidal tract signs—could not be observed in our patients.

So far, there is no definitive therapy for SH. The onset of symptoms after bleeding is reported in the literature as 4 months–37 years [1–3, 20]. The therapy consists primarily in finding possible causes of bleeding and hemostate them and minimizing risk factors that increase the likelihood of bleeding in the sense of a lifestyle change is indicated anytime. River et al. showed a reduction of the iron in the cerebrospinal fluid (CSF) with the treatment of trientine (2 g/die), a chelating agent which is primarily used for the treatment of Wilson with intolerance to D-penicillamine. The drug can cross the blood–brain barrier and, according to the source, slowing down the disease [20].

The treatment with trientine is recommended to patients with a history of brain surgeries or known sources of bleeding, i.e., patients with the secondary form of hemosiderosis.

All patients in our case series were considered to have a primary form of SH because no source of bleeding could be determined. Therefore, none of our patients were treated with trientine.

In the current literature, there are no cases addressed with blood patching in the sense of individual therapy for SH, but in the treatment of intracranial hypotension with hemorrhagic spinal epidural fluid collections [22]. According to this hypothesis, one of our five patients was treated with a spinal epidural blood patch as an individual therapy approach. The individual treatment approach of a blood patch therapy presupposes annual follow-ups with cranial MRI scans. So, the patient underwent yearly MRI controls and chose to be implanted with a CI in 2014 due to the rapidly progressing HL. According to the patient and as shown in the audiometric measurements, the HL did not slow down by this therapy. In contrast, the other patients with profound hearing loss were already in need of a CI and as a result, this therapy became less ideal—as the artifact from the CI on the MRI images would have made it difficult to evaluate the effect of the therapy via imaging. It is currently not possible to make a statement about the therapy results of blood patching after only one described case. But as an alternative, especially with very slow progression of the disease and relatively stable hearing, blood patching therapy can also be considered, with annual MRI evaluation, as a wait and scan therapy.

Although a retrocochlear genesis of SH has previously been considered, Nadol et al. showed in a histopathological study the cochlear involvement manifested by degeneration of the organ of Corti, the spiral ligament and the stria vascularis [23]. Our intraoperative measurements also add support on primary failure of the neural structures within the cochlea in these patients. Preoperatively, no compound action potentials (CAP) were measured by any of the patients. The ECAP AGF slope can be seen as a measure of the density of spiral ganglion cells [24]. Interestingly, we found in our cases a shallow ECAP AGF slope, which may be interpreted as low density of spiral ganglion cells (SGC). Or, in other cases, we did not succeed retrieving ECAP responses at all before the patient complained excessive loudness. This may also be attributed to low SGC density since responses of many SGNs are necessary to record an evoked potential.

Different outcomes in patients with SH using CI are described in the literature review of Taylor et al. as well as observed in the case series of seven patients of Modest et al. [14, 16]. In our case series, three patients showed sustained benefit from their CI in the mean follow-up time of 12 months. The outcome improved on average 26% in FMWT at 65 dB SPL. One patient gained no benefit and one showed initial improvement with subsequent decline in speech perception over time so that the progressive nature of the disease was clearly shown. While this patient achieved initially 70% in the FMWT at 65 dB SPL after the first CI on the left side in 2009, six months postoperatively, this value dropped to 45% in 2014 and then to 30% in 2018 in the same ear (Table 1). At least a comparison between the speech perception outcomes of the SH patients with CI with the results of our annual clinic report of the performed implantations between 2014 and 2018 is important. It was difficult to provide a long-term follow-up of the presented cases in this study. Some of our patients were lost to follow-up due to health problems caused by SH. The difficulty here is that a low rate of adaptation of the cochlear implants, especially in the case of a progressive disease, can lead to poorer hearing performance. In turn, poor hearing performance can lead to a low level of acceptance for wearing the CI. There are four groups that were categorized according to the preoperative result in the FMWT in our annual report of which we could compare two groups to the case series of SH patients [25]: adult patients with preoperative speech test results of 0% monosyllable at 65 dB and 5–20% monosyllable at 65 dB with their own HA achieved a median of 60% and 65% monosyllable at 65 dB, one year after implantation.

In case 1, there was no speech understanding at all. In such cases, auditory brainstem implant (ABI) or auditory middlebrain implant (AMI) should be considered as an alternative therapy option for the patient. Knowing that the SH has a retrocochlear genesis, it is important to verify where the damage in the auditory pathway is. Therefore, an audiological and radiological individual diagnosis for each patient is very important. In the future it will be very interesting to localize the damage exactly in the MRI to see how this affects the language understanding after receiving CI. It will also be interesting to verify prognostic factors due to the preoperative MRI.

In summary, it should be noted that early MRI diagnosis in the context of a rapid progressive bilateral SNHL within years, especially with a history of head trauma, aneurysm, neoplasm and neurosurgical procedures is indispensable. If SH is diagnosed in the imaging frequently, follow-ups should be timed to be able to assess the progression of the disease specially the progression of the leading symptom of HL. Early detection and intervention for HL can have an impact on the outcome with CI. Since the different outcomes with CI in patients with SH are uniform throughout the literature, they have to be included in the preoperative patients counseling.

## Data Availability

Not applicable.
